# Assessing Susceptibility of Debris Flow in Southwest China Using Gradient Boosting Machine

**DOI:** 10.1038/s41598-019-48986-5

**Published:** 2019-08-29

**Authors:** Baofeng Di, Hanyue Zhang, Yongyao Liu, Jierui Li, Ningsheng Chen, Constantine A. Stamatopoulos, Yuzhou Luo, Yu Zhan

**Affiliations:** 1 Institute for Disaster Management and Reconstruction, Sichuan University-Hongkong Polytechnic University, Chengdu, Sichuan 610200 China; 2 Department of Environmental Science and Engineering, Sichuan University, Chengdu, Sichuan 610065 China; 3 Institute of Mountain Hazards and Environment, Chinese Academy of Sciences, Chengdu, Sichuan 610041 China; 4 Stamatopoulos and Associates Co. and Hellenic Open University, Athens, 11471 Greece; 5 Department of Land, Air, and Water Resources, University of California, Davis, California 95616 United States; 6 Sino-German Centre for Water and Health Research, Sichuan University, Chengdu, Sichuan 610065 China; 7 Medical Big Data Center, Sichuan University, Chengdu, Sichuan 610041 China

**Keywords:** Natural hazards, Geomorphology

## Abstract

A gradient boosting machine (GBM) was developed to model the susceptibility of debris flow in Sichuan, Southwest China for risk management. A total of 3839 events of debris flow during 1949–2017 were compiled from the Sichuan Geo-Environment Monitoring program, field surveys, and satellite imagery interpretation. In the cross-validation, the GBM showed better performance, with the prediction accuracy of 82.0% and area under curve of 0.88, than the benchmark models, including the Logistic Regression, the K-Nearest Neighbor, the Support Vector Machine, and the Artificial Neural Network. The elevation range, precipitation, and aridity index played the most important role in determining the susceptibility. In addition, the water erosion intensity, road construction, channel gradient, and human settlement sites also largely contributed to the formation of debris flow. The susceptibility map produced by the GBM shows that the spatial distributions of high-susceptibility watersheds were highly coupled with the locations of the topographical extreme belt, fault zone, seismic belt, and dry valleys. This study provides critical information for risk mitigating and prevention of debris flow.

## Introduction

Debris flows are destructive mass movement, causing extensive economic losses and casualties around the world^1–3^. China is one of the mostly affected countries by debris flows, and approximately 50 thousand debris-flow sites distributed over 48% of the territory area of China^4,5^. These debris-flow sites are concentrated in Southwest China, particularly in Sichuan Province, Yunnan Province, and Tibet Autonomous Region. During 2000–2016, debris flows caused 90 deaths annually in Sichuan Province, which was twice as many as that caused by landslides^6^. Many earthquakes have happened in Sichuan, leading to massive debris flows^7^. Moreover, the magnitude and frequency of debris flows showed an increasing trend due to intensified environmental change and human activity^8–11^. The uncertainty of debris flows restricts the land-use planning and results in devastating effects on downstream areas. Susceptibility modeling is considered as the initial step towards hazard and risk assessment of debris flows, and it can also be used for debris flows warning system and environmental impact assessment. Therefore, it is indispensable to assess the susceptibility and identify the important factors associated with occurrence of debris flows for refining disaster management practices.

The methods for modeling susceptibility of debris flows vary widely among different countries or regions, which were generally categorized into physical models and statistical models^4^. The physical models simulate the dynamic process of landslides or debris flows based on physical mechanisms which consider hydrological conditions, slope stability, and soil strength decrease associated with landslide and debris-flow initiation^12,13^. The physical models are commonly employed for small-scale studies by using geographic information system and/or Monte Carlo simulations^14–16^. On the basis of the mechanisms of slope failure, physical models analyzed the dynamic process of debris flows and the hydrological conditions. To estimate the safety factor for a specific unit, the physical models require a wide range of small-scale data regarding mechanical soil characteristics and triggering factors, such as the potentially maximum volume of debris flows, 24-hour maximum rainfall, watershed cutting density, height difference, sediment concentration and population densities^17^. Due to the high data requirement, the physical models are not applicable for large-scale studies, but can be used to qualitatively validate statistical model results.

On the other hand, statistical models are less data-intensive and more suitable for simulating regional debris-flow susceptibility^18,19^. Parametric statistical models are commonly employed, such as analytic hierarchy process, logistic regression, and information value method, to link the regional susceptibility of debris flows to the potentially influencing factors^20^. In general, the factors include topographic geology, hydrometeorology, and human activities^21^. While the data categories are diverse, the data are all relatively easily retrieved through the Geographic Information Systems (GIS) and/or Remote Sensing (RS). For instance, the power-function model generates accurate and feasible estimates of debris-flow susceptibility in Yunnan, Southwest China^22^. A model comparison study found that the logistic regression model performed better than the physical models at regional scale^12^. While the parametric statistical models played an important role in simulating debris flows, they were inadequate to capture complex relationships that were difficult to be specified^23^. As a result, the prediction accuracy would be restrained.

Machine learning is a sophisticated statistical approach to modeling complex relationships between predictor and response variables, which is critical for assessing susceptibility of debris flows^24,25^. Machine learning approach, which pertains to the algorithmic modeling culture, learns model structures from training data and generally shows better predictive performance than parametric statistical models, such as logistic regression models^26,27^. Machine learning algorithms have shown great success in modeling disasters, such as landslides^28,29^, floods^30,31^, and debris flows^24^. Several popular machine learning algorithms, such as neural networks^23,32^, support vector machine (SVM)^33^, and naïve Bayes^34^, showed reliable performance in predicting occurrences of debris flows. Compared with the aforementioned machine learning algorithms, gradient boosting machines (GBM) generally showed better predictive performance in a series of model comparisons^27^. By utilizing the strengths of classification/regression trees and boosting, GBM grows a series of weak decision trees in a stage-wise fashion in order to slowly but steadily achieve optimization^35–37^.

This study aims to model the susceptibility of debris flows by watersheds in Sichuan, Southwest China to advance the management of risks related to debris flows. We compiled the data of debris-flow events for almost 70 years (1949–2017) in Sichuan, as well as a comprehensive predictor dataset. A sophisticated GBM model was developed to predict the susceptibility of debris flows by watershed units. The predictive performance of GBM was compared with four benchmark models, including the Logistic Regression (LR), the K-Nearest Neighbor (KNN), the Support Vector Machine (SVM), and the Artificial Neural Network (ANN). On the basis of the finely trained GBM model, the important predictor variables were identified, and the spatial distributions of debris-flow susceptibility were mapped. The results of this study are expected to provide a solid basis for predicting debris-flow disasters in the future, early warning, and risk prevention.

## Results and Discussion

### Predictive performance

**Figure 1:**
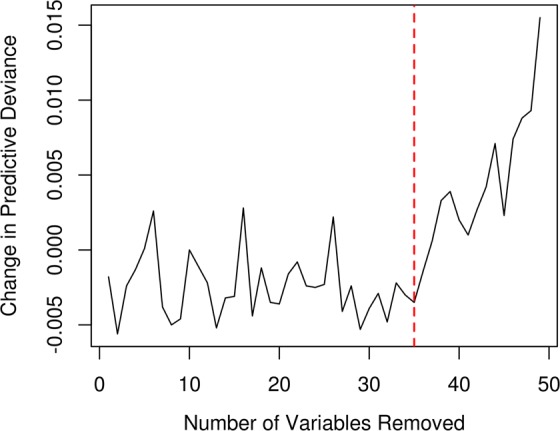
Change in predictive deviance during the stepwise removal of the least important variable from the gradient boosting machines (GBM). The dashed red line indicates the setting for the final GBM, which achieves quasi-optimal performance with much fewer variables.

In the cross-validation, the final GBM model showed good performance in predicting the susceptibility of debris flows, with the AUC of 0.88 and accuracy of 82.0% (Table 1). The prediction accuracy for the watersheds without debris-flow observations (85.4%) was relatively higher than that for the watersheds with debris flow observed (73.5%). It thus indicated that the prediction tended to biased towards the low susceptibility of debris flow. As the important hyperparameters in the GBM model, the number of trees and the tree depth were tuned to be 700 and 10, respectively. The hyper-parameter tuning process was essential for improving the predictive performance of the GBM model. The final GBM model retained 37 of the 72 predictors in the initial GBM model through the variable selection process, during which the prediction deviance initially fluctuated and then increased dramatically after 35 iterations (Fig. 1). The operation of variable selection reduced the data requirement and avoided spurious details in estimating the susceptibility of debris flow. Due to the difficulty in data collection, the debris-flow events were compiled from multiple sources over the long-term span (1949–2017). As this study focused on the spatial pattern of debris flow, the effects of data-source inconsistency were assumed to be negligible. The GBM model was superior to the benchmark models (i.e., LR, KNN, SVM, and ANN) in predicting the susceptibility of debris flow (Table 2). For the KNN model, the best predictive performance was achieved when the number of neighbors considered equaled to 15. For the SVM model, the kernel, gamma, and cost of constraints violation were tuned to radial, 0.01, and 10, respectively. For the ANN model, the number of units in the hidden layer was set to 3, and the decay was set to 0.1. The previous studies also found that GBM models exhibited better performance in simulate susceptibility of debris flows than SVM and mixture discriminant analysis did, although the research domains of these studies were distinctive^33,34^. In the future, more comprehensive model comparisons will be necessary to guide the model selection for simulating debris flow.

**Table 1:** Observation vs. prediction of watershed-based debris flow in Sichuan by gradient boosting machine^a^.

	Observation-N	Observation-Y	Accuracy (%)
Prediction-N	1514	259	85.4
Prediction-Y	186	515	73.5
Total	1700	774	82.0

^a^N means there did not exist debris flow, and Y represents there existed debris flow.

**Table 2:** Performance comparison of Gradient Boosting Machine (GBM), Logistic Regression (LR), K-Nearest Neighbor (KNN), Support Vector Machine (SVM), and Artificial Neural Network (ANN) in predicting susceptibility of debris flow based on cross-validation.

Metric	GBM	LR	KNN	SVM	ANN
Accuracy (%)	**82**.**0**	79.5	78.6	81.8	80.5
AUC^a^	**0**.**88**	0.85	0.83	0.86	0.86

^a^AUC: Area under curve (AUC) of receiver operating characteristic (ROC), with the best performance shown in **bold**.

### Important predictor variables

**Figure 2:**
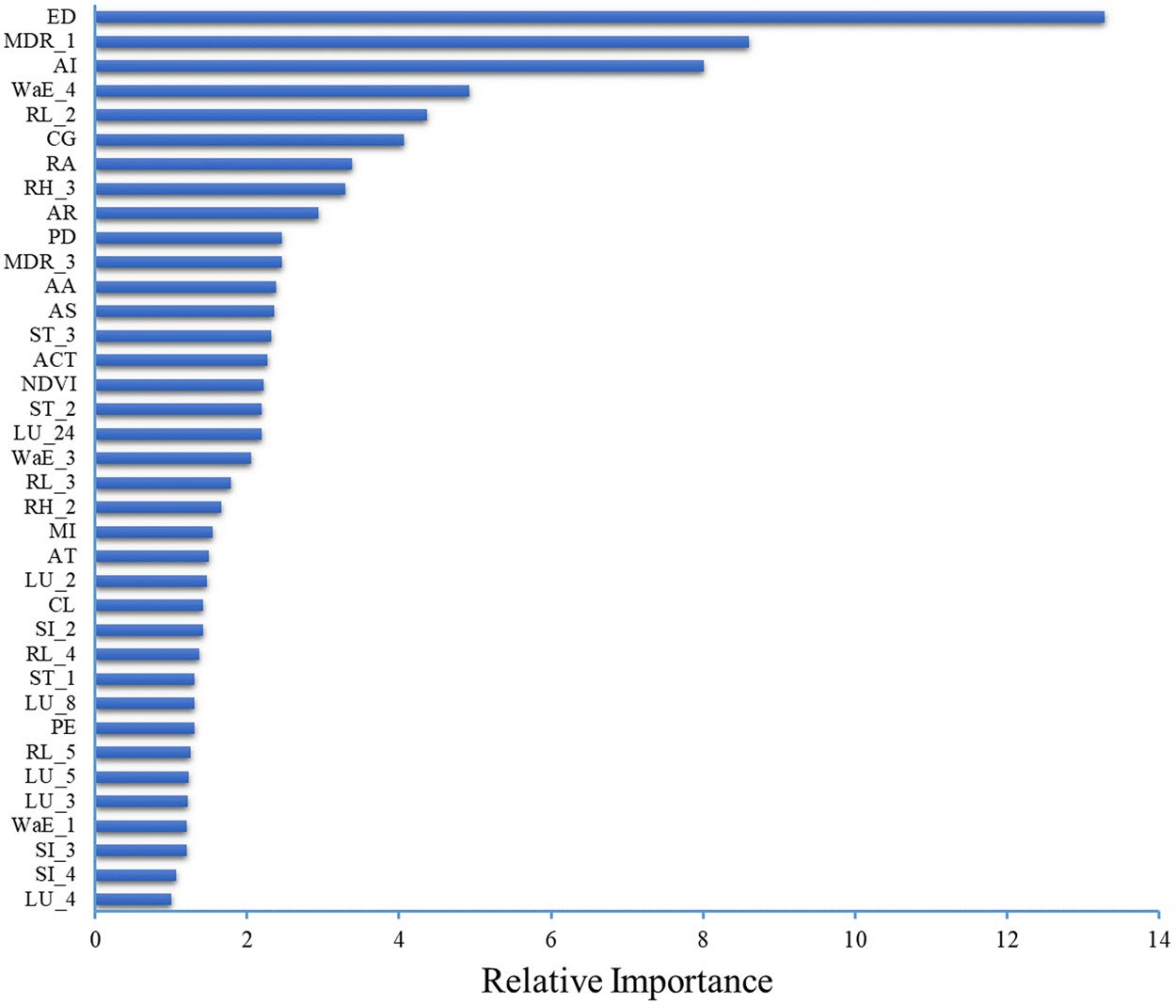
Variable importance plot for the gradient boosting machine predicting the susceptibility of debris flow in Sichuan. The relative importance is normalized so that they sum up to 100 for more intuitive interpretation. Please refer to Table 4 for the description of the variable acronyms.

The elevation range was the most important predictor variable in the final GBM model, with the importance value of 13.3, and the associated predictor variable of channel gradient exhibited an importance value of 4.1 (Fig. 2). The elevation range plays a critical role in the formation of debris flow by determining the level of potential energy. Larger elevation difference leads to higher potential energy, creating favorable conditions for debris flows. The debris flow mainly occurred in the mountainous areas, as well as the surroundings of undulating plateau^38,39^. A previous study found that debris flow tended to happen when the height difference reached more than 300 m^38^. In our study area, more than 97% of the river basins in the valley where debris flow happened, had a height difference ranging from 400 to 4000 m. In addition, channel gradient provided the conditions for the conversion of loose material forces in the watershed into kinetic energy. It has been acknowledged that higher channel gradient favored occurrence of debris flow^40^.

The maximum daily rainfall was the second most important predictor variable, with the importance value of 8.6, while the importance values of the annual rainfall and the maximum 3-day rainfall were 2.9 and 2.5, respectively (Fig. 2). Rainfall is one of the essential trigger factors of debris flow^41,42^. Heavy rainfall indicated by maximum daily rainfall tend to trigger debris flow when source materials are abundant. The maximum 3-day rainfall with a longer time span is supplementary to the maximum daily rainfall. The annual rainfall together with the aridity index reflect the dry-wet condition in the long term.

The aridity index, with the importance value of 8.0, was the third most important predictor variable (Fig. 2). Extremely arid climates have been found to be highly associated with occurrences of debris flows, which are usually caused by extremely dry periods followed by wet seasons^43,44^. The drought background or the dry-wet alternating climate conditions aggravate soil cracks, change the structure/composition of soil, and lower the rainfall thresholds triggering debris flows. Drought degraded vegetation cover, weakens soil structure, and increases loose solid materials prone to debris flows due to their distribution of varied debris and disturbed soil^45–47^. Debris flows were found to occur on the sunny side more frequently than the shady side of a mountain, suggesting that the hydrothermal conditions, particularly droughts, influenced occurrences of debris flows^47^.

The water erosion intensity and the negative effects of anthropogenic activity were also important factors to the susceptibility of debris flows. As indicated above, Sichuan lies in the transition area between the Qinghai-Tibet Plateau and the plain region. The previous studies showed that the soil erosion was 0.5–7 mm/y in the Qinghai-Tibet Plateau from 30 Ma (million anniversary) ago to the present^47–49^. While the rock/soil types play a critical role in the formation and accumulation of surface sediments, the rapid soil erosion provides massive unconsolidated materials which is source material for debris flows. Earthquakes induce secondary disasters such as landslides providing debris flows with source materials, and the impact was indicated by the seismic intensity. In addition, the anthropogenic activities such as road construction and land overexploitation accelerate soil erosion and consequently exacerbate debris flow^50^, which is reflected by the high importance of the national road length, the number of settlement sites, and the population density (Fig. 2).

As the predictor variables with respect to soil types, the area proportions of clay, silt, and sand exhibited relatively negligible importance to the susceptibility of debris flow, with importance values of 2.3, 2.2, and 1.3, respectively. The soil types directly affected the sediment concentration of debris flow, which in turn influenced its size and flow state. The clay content influences the formation of debris flow by affecting the initiation of debris flow, especially for viscous debris flow^51^. A moderate amount of clay content was an essential precondition for forming large-scale debris flow with a high amount of sediment concentration. Under the effect of precipitation, the loose clay soil expands after water absorption, leading to an increase in pore pressure and failure of viscous resistance, which accelerated the formation of debris flow.

The effects of factors influencing debris flow formation were complicated by non-linearity and interactions. It was therefore very important to identify the key controlling factors. According to the present study modeling the susceptibility of debris flows in Sichuan Province, topographic conditions, geological background, precipitation, and anthropogenic activities played an important role in the formation of debris flow. In addition, the susceptibility of debris flow was also associated with the drought conditions, road construction, soil types, and land use, which were indispensable factors in evaluating the susceptibility of debris flow at regional level.

### Susceptibility mapping

**Figure 3:**
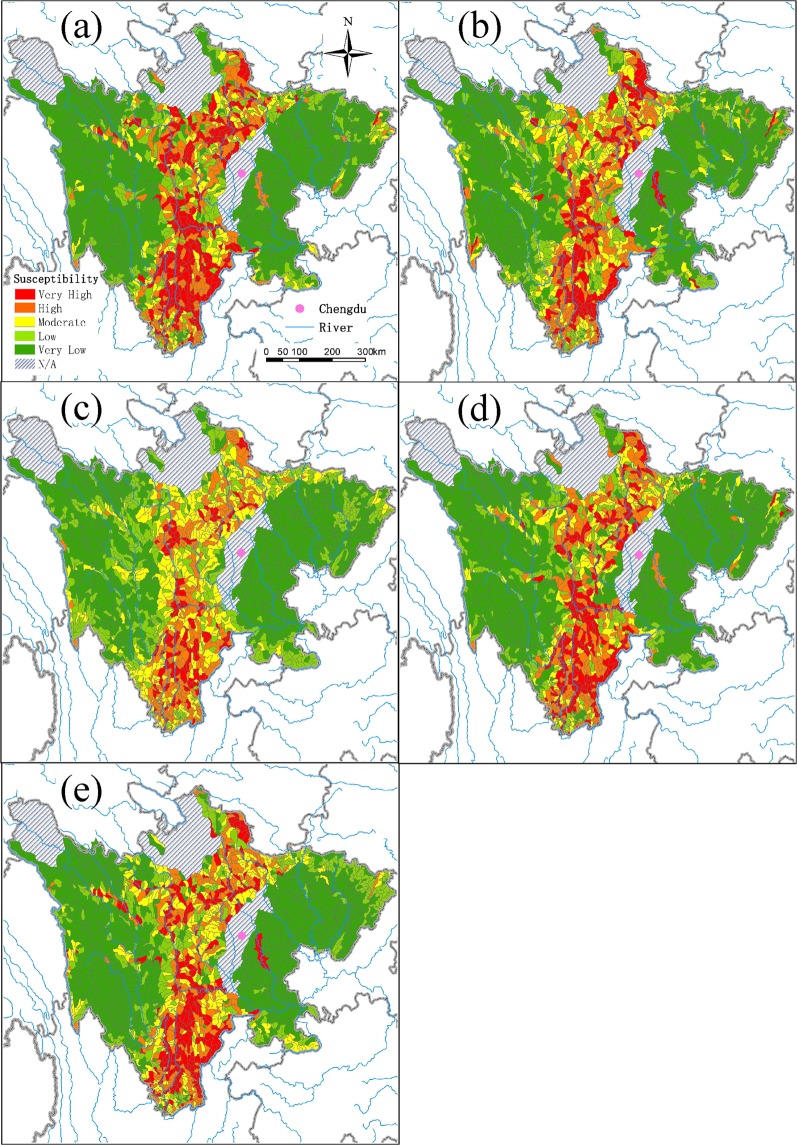
Spatial distributions of the predicted susceptibility of watershed-based debris flow by using (**a**) gradient boosting machine, (**b**) logistic regression, (**c**) K-nearest neighbor, (**d**) support vector machine, and (**e**) artificial neural network. N/A: Not applicable. The debris-flow formation conditions were inadequate in the plateaus and plains, and thus these areas were excluded from the susceptibility modeling.

As a result of the GBM model, the debris-flow map was constructed. It shows the spatial distribution of the susceptibility, which was classified into five categories, including very low, low, moderate, high, very high (Fig. 3a). Table 3 shows the areas and numbers of watersheds by susceptibility category. The watersheds of very low susceptibility occupy the largest area (226,600 km^2^), with the largest number of watersheds (1,342) accounting for the 47% of the study area. These watersheds were mainly distributed in western plateau and mountainous areas, as well as eastern plain and hilly areas. The number of the moderate-susceptibility watersheds is the smallest (212), and the area is the smallest (33,500 km^2^; 7% of the study area). The watersheds with high or very high debris-flow susceptibility (110,100 km^2^), accounting for 22% of the total areas, are mainly distributed in the central mountainous region across Sichuan from north to south. These areas are located in the lower reaches of the Yalong River and the Dadu River, and the upper reaches of the Minjiang River near the Wenchuan earthquake. The susceptibility map of watershed-based debris flow evaluated by GBM was considerably different from those evaluated by the benchmark models (Fig. 3). The number of watersheds with very-high susceptibility was largest as predicted by GBM (297), followed by ANN (243), SVM (234), LR (194), and KNN (130). The watersheds with high or very-high susceptibility predicted by GBM were more concentrated near the Wenchuan earthquake region compared with the predictions made by the other models. In addition, the areas with moderate susceptibility were overestimated by KNN, which also underestimated the areas with very-low or very-high susceptibility, manifesting that KNN did not perform well in mapping the susceptibility of debris flow. The map predicted by using the GBM model qualitatively and quantitatively characterized the spatial distribution of the debris-flow susceptibility for the watersheds.

**Table 3:** Classification for the predicted susceptibility of watershed-based debris flow by using gradient boosting machine.

Class	Number of Watersheds	Area (km^2^)	Percentage (%)
Very low	1342	226600	47
Low	328	56500	12
Moderate	212	33500	7
High	292	51500	10
Very high	297	58600	12
N/A^a^	/	58000	12

^a^N/A: Not applicable. The debris-flow formation conditions were inadequate in the plateaus and plains, and thus these areas were excluded from the susceptibility modeling.

The western part of the study area was mainly located in the hinterland of the Qinghai-Tibet Plateau. The topography was dominated by plateaus and hill-shaped areas with gentle fluctuation. The environmental conditions in all areas, except for some deep-cut river valleys, were insufficient for development of debris flow, where the watersheds were dominated by the ones with very low susceptibility. The eastern part of the study area was mainly distributed in the Sichuan basin and hilly landforms, where the topography did not vary largely. Other than the watersheds of the moderate susceptibility in the Qujiang River basin, most of the watersheds in the eastern Sichuan were of low or very low susceptibility of debris flow.

**Figure 4:**
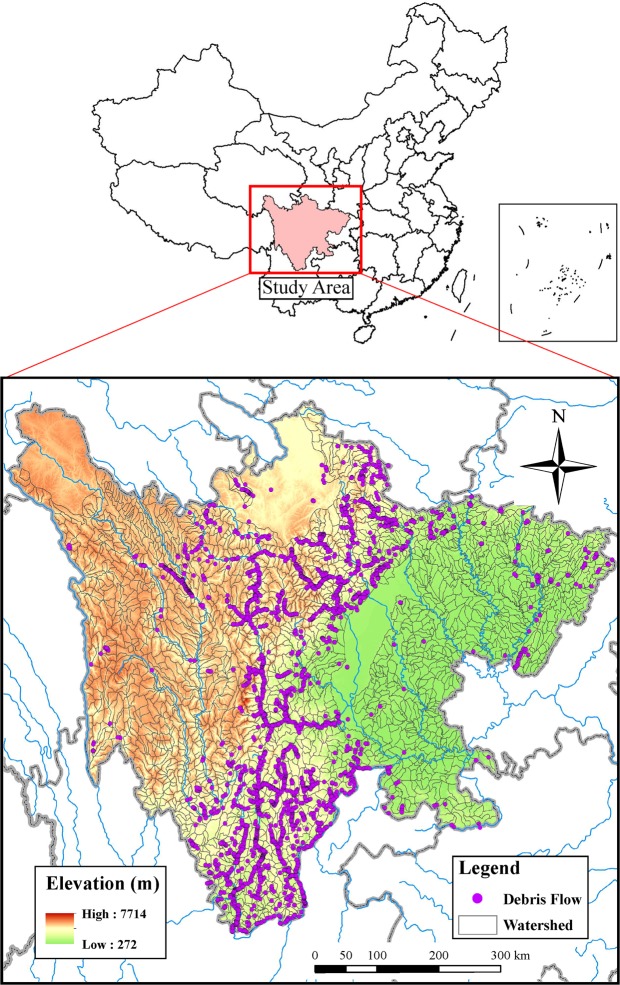
Map of the study area, watershed divisions, and locations of the observed debris flow.

The watersheds of high debris-flow susceptibility were mainly concentrated in the western part of the study area. Topographically, the highly susceptible areas were located in the topographic belt transiting from the Tibetan Plateau to the Sichuan Basin. In the Hengduan Mountains lying from north to south, the terrain is fragmented and the hills are steep, creating adequate conditions for debris flows. The fault zones of Longmenshan, Xianshuihe, and Anninghe distributed in an “Y” shape (shaded area in Fig. 4), which was generally consistent with the seismic zones. In these zones, earthquakes and rock fractures occurred frequently, with a number of secondary mountain disasters, providing abundant source materials to debris flows.

In addition, the high-susceptibility areas were coupled with the dry valley landscape in the study area. Among those areas, the Yalong River and its tributaries, including the Anning River Valley, the Dadu River, the upper reaches of Min River, middle and lower reaches of the Jinsha River, were the concentrated areas of debris flow, which were also identified as the areas with high or very high susceptibility of debris flow. The dry valleys with fragile ecosystem and severe soil erosion were found in all the rivers of Yalong, Dadu, Min and Jinsha. The dry valleys were affected by local circulation and forming activity. The evaporation in the valleys was far greater than the precipitation, where the vegetations were hard to grow and the soil erosion was severe. Moreover, in the dry valleys, inappropriate cultivation, such as steep slope reclamation and smooth slope cultivation, led to severe gravity erosion prone to formation of debris flow. Meteorologically, heavy rains tended to trigger debris flows in these areas. In addition, the construction of roads and hydropower stations was intensive in these areas and tended to aggravate the susceptibility of debris flows.

In general, the spatial distribution of high susceptibility of debris flow in Sichuan Province had a degree of overlap with the topographical extreme belt, fault zone, seismic belt, and dry valleys. Prevention and control of debris-flow risk in the study should be focused on these four types of highly coupled areas for preventing or mitigating sudden mass deaths caused by debris flow. We studied the spatial distribution of debris flow for the watersheds in Sichuan Province, and clearly identified the critical areas for the monitoring and early warning of debris flow. The results had very important practical significance and social benefits for disaster prevention and reduction.

## Conclusions

On the basis of the comprehensive dataset associated with debris flows, a GBM model was developed to simulate the susceptibility of debris flows in Sichuan, Southwest China. The GBM model showed highlighted predictive performance by adequately capturing the complex relationships between the predictor and response variables, which was superior to the benchmark models (i.e., LR, KNN, SVM, and ANN). The elevation range, maximum daily rainfall, and aridity index were identified as the most important predictor variables influencing the occurrences of debris flows, which provided invaluable information for management. In addition, the high intensity area of water erosion, length of national roads, channel gradient, and number of settlement sites also played an important role in the susceptibility of watershed-based debris flow. The susceptibility map was produced by using the GBM model. This map could facilitate initial hazard evaluation for development planning. The spatial distributions of the high-susceptibility watersheds were highly coupled with the locations of the topographical extreme belt, fault zone, seismic belt, and dry valleys. It is essential to conduct monitoring and risk prevention in the highly susceptible areas.

## Materials and Methods

### Study area

The study area, i.e., Sichuan Province, is located in Southwest China (26°03′–34°20′N, 97° 22′–110°10′E), covering an area of approximately 485 thousand km^2^ (Fig. 4). The complex landform of Sichuan is dominated by mountainous and hilly lands which account for 85% of the total terrain. The main part of Sichuan lies in the geomorphological transition area between the Tibetan Plateau and the Middle-Upper Yangtze River Plain, with elevation differences larger than 4000 m. Sichuan is mainly of monsoon climate, and approximately 70% of the annual average rainfall (around 1000 mm) happens from June to September. The major rivers in Sichuan, including the Yalong River, the Minjiang River, the Tuojiang River, the Jialing River, and the Wujiang River, are tributaries of the Yangtze River. The stratums of Sichuan were well developed from the Upper Archean to the Quaternary. The species of magmatic rocks are abundant, and granites account for the major proportion of the rocks. Being divided by the Longmenshan fault zone, the western and eastern Sichuan show large differences in terrain, stratigraphic structure, and meteorological conditions. Sichuan Province is a highly active seismic zone, where three major earthquakes happened in the last ten years, including the Wenchuan M_s_ 8.0 earthquake in 2008, the Lushan M_s_ 7.0 earthquake in 2013, and Jiuzhaigou M_s_ 7.0 earthquake in 2017^52^. Similar to the previous studies^1,53^, the susceptibility of debris flows was modeled by watersheds, which are basic units for the whole phenomenon of debris flows, which includes triggering, propagation, and stoppage^38^. On the basis of the digital elevation model (DEM), streamline map, and satellite images, we delineated 2474 watersheds by using both the automatic and manual vectorization methods (Fig. 4).

### Data preparation

A total of 3839 debris-flow events were identified in 774 watersheds of Sichuan during 1949–2017. The debris-flow data from 1949 to 2004 were obtained from the Sichuan Geo-Environment Monitoring Program^6^, and the debris-flow events during 2005–2017 were compiled from news reports and literatures. The locations of the debris flows were concentrated in the mid-western Sichuan, where a considerable number of population dwell (Fig. 4). The spatial distributions of the debris-flow events generally coincided with the arid valley extending from the Hengduan Mountains in the Eastern Tibetan Plateau to the Yuannan-Guizhou Plateau. As debris flows were rarely observed in the plateau and plain areas, this study focused on the watersheds located in the mountainous and hilly areas. The watershed with/without debris flow occurred were labelled as presence/absence of debris flow for the subsequent modeling.

**Figure 5:**
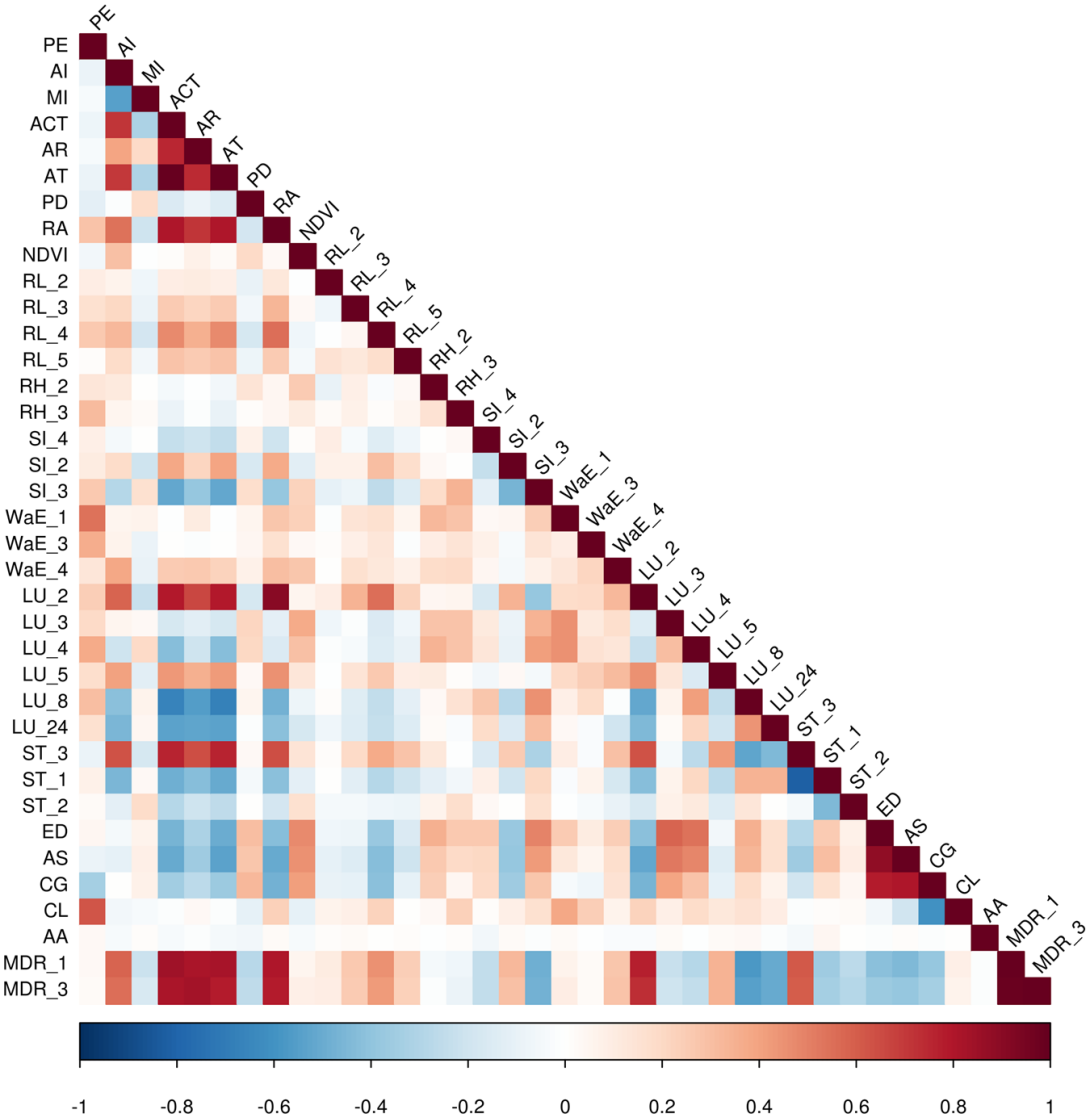
Correlations among the predictor variables of the final gradient boosting machine. The correlations were evaluated by using the Spearman correlation coefficient. Please refer to Table 4 for the description of the variable acronyms. The color of each grid cell represents the correlation strength (annotated on the bottom bar) of the two variables labelled in the leftmost and topmost ends.

According to the present knowledge on debris flows and data availability, 72 predictor variables were determined for modeling the susceptibility of debris flows by watersheds (Table 4). The geomorphological factors, including the area, perimeter, elevation difference, channel gradient, average slope, average aspect, and channel length were derived from the DEM dataset (30 m resolution) retrieved through the Advanced Spaceborne Thermal Emission and Reflection Radiometer^54^. The geological factors, including the length of active faults and the type of seismic intensity (at 1:4000000 scale), were obtained from the China Seismic Information^55^. The rock hardness was rasterized from the 1:200000 lithological composition map of Sichuan^55,56^. The meteorological conditions, including the annual average rainfall, annual average temperature, annual accumulated temperature above 10 °C, aridity index, and moisture index, were acquired from the corresponding raster files (500 m resolutions) published in the Data Center for Resources and Environmental Sciences (RESDC) of the Chinese Academy of Sciences^57^. The maximum daily rainfall and the maximum 3-day rainfall were derived from the daily observations at meteorology sites^58^. The Normalized Difference Vegetation Index (NDVI; 300 m spatial resolution) were derived from the Proba-V satellite retrievals^59^. The land use types, population densities, soil erosion intensity, and soil textures were obtained from the RESDC^57^. The lengths of county roads, highways, and railways were summarized from the OpenStreetMap for each watershed^60^. The locations of settlement sites were obtained from the Socioeconomic Data and Applications Center (SEDAC)^61^. The values of the following predictor variables are discretized: seismic intensity, rock hardness, soil texture, water erosion intensity, wind erosion intensity, freeze-thaw erosion intensity, land use, and road length. The raw data of the predictor variables were preprocessed to the delineated watersheds by using various tools in the ArcGIS, including Calculate Geometry, Zonal statistics as Table, Spatial Join, Tabulate Intersection, Raster Calculator, Surface, Reclassify, Buffer, and Kriging Interpolation. The correlations between the predictor variables were evaluated with the Spearman correlation coefficients (Fig. 5).

**Table 4:** Summary of predictor variables for developing the classification models.

Variable	Acronym	Unit	Reference
Debris flow events	DF	—	^6^
Area	Area	m^2^	^54^
Perimeter	PE	m	^54^
Elevation difference	ED	m	^54^
Channel gradient	CG	%	^54^
Average slope	AS	°	^54^
Average aspect	AA	°	^54^
Channel length	CL	km	^54^
Active fault	AF	m	^55^
Seismic intensity	SI	—	^55^
Rock hardness	RH	—	^55, 56^
Aridity index	AI	—	^57^
Moisture index	MI	—	^57^
Annual accumulated temperature above 10 °C	ACT	°C	^57^
Annual rainfall	AR	mm	^57^
Annual temperature	AT	°C	^57^
Maximum daily rainfall	MDR_1	mm	^58^
Maximum 3-day rainfall	MDR_3	mm	^58^
Normalized Difference Vegetation Index	NDVI	—	^59^
Soil texture	ST	—	^57^
Water erosion intensity	WaE	—	^57^
Wind erosion intensity	WiE	—	^57^
Freeze-thaw erosion intensity	FtE	—	^57^
Land use	LU	—	^57^
Settlement sites	RA	—	^61^
Population density	PD	persons/km^2^	^57^
Road length	RL	km	^60^

*Detailed classification of part indexes.

Seismic intensity: (1) < VI; (2) VI; (3) VII; (4) VIII; (5) ≥ IX.

Rock hardness: (1) Very strong; (2) Strong; (3) Medium; (4) Weak; (5) Very weak; (6) Solum.

Soil texture: (1) Sand; (2) Silt; (3) Clay.

Water erosion intensity: (1) Very low; (2) Low; (3) Moderate; (4) High; (5) Very high; (6) Extreme.

Wind erosion intensity: (1) Very low; (2) Low; (3) Moderate; (4) High; (5) Very high; (6) Extreme.

Freeze-thaw erosion intensity: (1) Very low; (2) Low; (3) Moderate; (4) High.

Land use: (1) Paddy field; (2) Dry farm; (3) Forest; (4) Shrubbery; (5) Open forest; (6) Other forest; (7) High coverage grassland; (8) Moderate coverage grassland; (9) Low coverage grassland; (10) River; (11) Lake; (12) Reservoir; (13) Permanent glacier; (14) Mudflat; (15) Bottomland; (16) Urban land; (17) Rural residential area; (18) Other construction land; (19) Sand; (20) Gobi; (21) Saline-alkali soil; (22) Wetland; (23) Bare; (24) Rock; (25) Others.

Road length: (1) Highway; (2) National road; (3) Provincial road; (4) County road; (5) Railway.

### Model description

For simulating the susceptibility of debris flow (i.e., occurrence probability) by watersheds, a GBM model was trained to minimize the following loss or deviance function^62^:

1 $$L(y,f(x))=\sum \{\,\mathrm{log}(1+\exp (f({x}_{i}))-{y}_{i}f({x}_{i})\}$$

where *x* represents the predictor variables (Table 4), *y* is the observation of debris flow event (i.e., occurrence/non-occurrence), and *f*(*x*) is the GBM model parameterized through the following procedure^35,36^:

2 $${\rm{Model}}\,{\rm{initialization}}:\,{f}_{0}(x)=\,\mathrm{log}\,\frac{\sum {y}_{i}}{\sum (1-{y}_{i})}$$

For *k* = 1 to *K*, repeat the steps below in order to obtain *f*_*K*_(*x*):

- Draw a subsample from the training dataset at random without replacement
- Use the model updated at step *k*-1 to calculate the residuals (${\tilde{y}}_{j}$) for this sub-sample:

3 $${\tilde{y}}_{j}={y}_{j}-\frac{1}{1+\exp (\,-\,{f}_{k-1}({x}_{j})}$$

- Develop a new classification tree *ρ*_*k*_ to fit ${\tilde{y}}_{j}$
- Update the model by adding the fitted tree with a shrinkage rate (default: *λ* = 0.05):

4 $${f}_{k}(x)={f}_{k-1}(x)+\lambda {\rho }_{k}$$

The model output was the occurrence probability or susceptibility of debris flow.

Hyperparameter tuning and variable selection were performed to further refine the GBM model. The values of the hyperparameters, including the number of trees (*K*) and the tree depth, were determined when the associated prediction deviance reached the minimum in the 10-fold cross-validation (explained in the next subsection). Similarly, the predictor variables of the GBM model (initially 72 variables) were selected by using the backward selection strategy, where the least important variable (explained in the next subsection) was removed from the model one at a time. The set of predictor variables with the lowest prediction deviance in the cross-validation was selected to build the final GBM. The R packages of *gbm* and *dismo* were used for training the GBM model and making predictions^62,63^. R package *doParallel* was used to run the modeling process in a parallel manner for reducing the computing time^64^.

With the same data and predictor variables, the GBM model was compared with four benchmark models, including LR, KNN, SVM, and ANN, to evaluate the performance in predicting the susceptibility of debris flow. LR is a generalized linear model for classification parameterized by the maximum likelihood. KNN, a non-parametric algorithm, groups *K* samples nearest to a particular sample into the same category, and the prediction is the mode in this category. SVM classifies samples in feature spaces by hyperplanes based on maximal margin classifiers, and kernels are applied to expand the feature spaces for accommodating non-linear boundaries. ANN, a kind of adaptive system with multi-layer neurons, learns from the pre-provided input and output data. The LR, KNN, SVM, and ANN models were implemented with *R* packages of *stats*, *class*, *e1071*, and *nnet*^65–67^, respectively. All the parameters in GBM, KNN, SVM, and ANN models were tuned through the grid search method.

### Model evaluation

The model predictive performance was evaluated with the commonly used metrics, including the prediction accuracy and the area under curve (AUC) of the receiver operating characteristic (ROC). The AUC illustrated the changes of true positive rate and false positive rate when the discrimination threshold varied. The 10-fold cross-validation approach was employed to obtain model predictions, where the training and prediction data were separated in order to reflect more realistic performance. Specifically, the training dataset was randomly partitioned into 10 similarly-sized groups. At each of 10 rounds, 9 groups were used to train the model which made predictions for the remaining group. After 10 rounds, every observation was paired with a prediction value.

In addition, the variable importance measure, which is valuable for interpreting and diagnosing the GBM model^35^, was used to evaluate the effects of the predictor variables on the susceptibility of debris flows. The variable importance was indicated by the mean decrease in deviance resulted from the splits on that variable. A partial dependence plot showed the effects of a predictor variable on the susceptibility of debris flows after subtracting the average effects of all the other predictor variables.

### Susceptibility mapping

The susceptibility of debris flow for each watershed in Sichuan was estimated by using the final GBM model and the benchmark models. The levels of susceptibility were divided into five classes, including very high, high, moderate, low and very low, based on the equal-interval classification method. Arc GIS was used to map the watershed-based susceptibility for intuitive visualization.
